# Current development of stretchable self-powered technology based on nanomaterials toward wearable biosensors in biomedical applications

**DOI:** 10.3389/fbioe.2023.1164805

**Published:** 2023-04-11

**Authors:** Qianqian Wang, Xu Sun, Chen Liu, Chunge Wang, Wenjie Zhao, Zehui Zhu, Sainan Ma, Sheng Zhang

**Affiliations:** ^1^ Ningbo Innovation Center, Zhejiang University, Ningbo, China; ^2^ State Key Laboratory of Chemical Engineering, College of Chemical and Biological Engineering, Zhejiang University, Hangzhou, China; ^3^ Faculty of Science and Engineering, University of Nottingham Ningbo, Ningbo, China; ^4^ School of Mechanical and Energy Engineering, NingboTech University, Ningbo, China

**Keywords:** self-powered, stretchable device, biosensor, biomedical applications, wearable

## Abstract

In combination with the growing fields of artificial intelligence and Internet-of-things (IoT), the innovation direction of next-generation biosensing systems is toward intellectualization, miniaturization, and wireless portability. Enormous research efforts have been made in self-powered technology due to the gradual decline of traditional rigid and cumbersome power sources in comparison to wearable biosensing systems. Research progress on various stretchable self-powered strategies for wearable biosensors and integrated sensing systems has demonstrated their promising potential in practical biomedical applications. In this review, up-to-date research advances in energy harvesting strategies are discussed, together with a future outlook and remaining challenges, shedding light on the follow-up research priorities.

## 1 Introduction

The demand for flexible biosensors in biomedical applications is rising rapidly, especially those integrated with self-powered technology (Zhang et al., 2021a; He et al., 2021; Ouyang et al., 2021; Zeng et al., 2022a). The biological system of humans is extremely complex and possesses a series of physiological signals. These signals provide essential information on the human body’s health status (Zhang et al., 2021b; Liu et al., 2021; Zhang et al., 2022a; Jackson et al., 2022; Kukkar et al., 2022). Biosensors’ accuracy, flexibility, stretchability, lightness, and portability have been much improved based on newly developed fabrication techniques and sensing technologies (Guo et al., 2021; Tiwari et al., 2022). However, miniaturized biosensors such as epidermal sensors, tattoo sensors, and tactile sensors usually present limited size and thickness (Gao et al., 2019; Wang et al., 2019; Liu et al., 2020). Therefore, power supply to these types of sensors is challenging since the size of the power device needs to be adapted to the miniaturized nature of the biosensor so that the integrated system can be wearable and stretchable (Table 1).

**TABLE 1:** Examples of self-powered technology towards wearable biosensors and biomedical applications. P(VDF-TrFe): poly (vinylidene fluoride-co-trifluoroethylene); CNTs: carbon nanotubes; PVDF: polyvinylidene fluoride; PT: polythiophene; MAPbBr_3_: Methylammonium lead tribromide; BTO: barium titanate; EGain: eutectic gallium-indium; Co-NPC: cobalt-based nanoporous carbon; PANI: polyaniline; NPCO: nanoporous cobalt oxide; PVA/PA: polyvinyl alcohol/phytic acid; TSCH: transparent, stretchable and conductive hydrogel; FEP: fluorinated ethylene propylene; CNTs: carbon nanotubes; rGO: reduced graphene oxide; MWCNTs: multi-walled carbon nanotubes; NQ: naphthoquinone; PET: Polyethylene terephthalate; PEI: polyethyleneimine; NCQDs: nitrogen-doped carbon quantum dots; OPs: organophosphorus pesticides; AFP: alpha-fetoprotein.

	Material	Modification/Functionalization/Fabrication		Application	Effectiveness	Ref
Piezoelectric nanogenerators	P(VDF-TrFe) nanofibers	Surface modified by ZnSnO_3_ and CNTs		Sensing of imperceptible pulse	High output power and high sensitivity	Kang et al. (2022)
	PVDF film	Promoted by PT		Detection of human motion	Prominent stability and sensitivity	Li et al. (2022)
		Coated with Ag and encapsulated with PT			Great endurance performance	Liang et al. (2022)
		Embedded with MAPbBr_3_ single crystals			Excellent output power and power density	Lee et al. (2022)
		Wrapped around the cylindric elastomer		Dynamic-static pressure detection and sports evaluation	Splendid open circuit voltage, short circuit current, and power density	Kong et al. (2022)
	Au-deposited elastomer film	Treated into a rotating square pattern		Monitoring of target muscle contraction and the corresponding joint	High sensor-to-sensor and in-sensor uniformities	Gu et al. (2021)
	(P(VDF-TrFE)	Applied entangled Ag nanowires as top electrode		Health-related monitoring	Great output performance and mechanical stability	Kim et al. (2021)
	PDMS film	Combined with BTO		Human joint bending motion monitoring	Satisfactory stretchability and excellent skin conformality	Yu et al. (2022b)
Triboelectric nanogenerators	EGaIn liquid metal	Embedded in silicone channel		Intelligent prosthetics and medical rehabilitation	Low detection limit, fast response time, and high stretchability	Peng et al. (2022)
	PVDF	Incorporated with Co-NPC		Human motion sensing	Excellent output performance and long-term stability	Qu et al. (2022)
	Ecoflex fiber	Coated with PANI		Sensing of glucose, creatinine and lactate acid in sweat	Multifunctional and high sensitivity	Zhao et al. (2022b)
	NPCO and MXene	Combined with silicone to form nanocomposite		Detection of foot pressure distribution	High power density, sensitivity, hydrophobicity, and water resistivity	Rahman et al. (2022b)
	PVA/PA hydrogel	Freeze-thaw cycle		Medical nursing HMI system	Excellent mechanical and electrical properties	Yang et al. (2022a)
Biofuel cells	TSCH	Facile photo-triggered gelation		Tactile sensing	High stretchability, great electrical conductivity, and transparency	Liu et al. (2022a)
	FEP film	Tightly attached to the electrode		Detection of rain water	Short circuit current, quick response time	Zeng et al. (2022b)
	Various waste textiles	4-finger knitting		Sensing units in sports facilities	Eco-friendly	Sahu et al. (2022)
	CNTs-rGO film	Simple hydrothermal process		Water sample analysis	Low detection limit and high deformability	Qin et al. (2022)
	MWCNTs	Integrated with NQ and lactate oxide		Hydrogen sensing	Good human compatibility, easy integration, and cost effectiveness	Yang et al. (2022b)
		Dropped on carbon fiber		Detection of lactate in sweat	Good stability	Yin et al. (2021)
	Carbon nanorods	Assembled by coral-like hierarchical meso-macroporous carbon			High sensitivity, low detection limit, and economical cost	Xu et al. (2022a)
	PET	Printed by Ag ink		Urine detection	Easy to collect urine	Su et al. (2022)
	CNTs	Combined with PEI and glucose oxidase		Glucose detection in urine	High power density, broad sensing range, and great interference ability	Zhang et al. (2021d)
Photovoltaics	MoS_2_ quantum nanosheets		Incorporated with PVDF	Power supply for wearable electronics	Remarkable electrical output, lightweight, and high flexibility	Nardekar et al. (2022)
	Zn_1-2x_ (Fe_x_Li_x_)O films		Low-cost sol-gel process		Low cost and switchable photoresponse	Gao et al. (2022)
	NCQDs		Incorporated with TiO_2_ nanoparticles and indium tin oxide	Detection of OPs in environment and food	Broad detection range and low sensing limit	Cheng et al. (2019)
	Perovskite NaNbO3		Deposited by Ag	Detection of cancer-related proteins	Rapid and accurate detection	Xu et al. (2022b)
	Carbon black		Incorporated with TiO_2_ and KuQ dye	Detection of ethanol	Broad sensing range and low detection limit	Mazzaracchio et al. (2022)
	FTO substrate		Deposited by NaYF4:Yb, ZnO, and CdS	Monitoring of AFP	Low detection limit and wide sensing range	Zhai et al. (2019)
Thermoelectric generator	Nickel doped Bismuth Telluride		Electrodeposition	Monitoring of temperature and humidity	High output voltage and power density	Toan et al. (2022)
	PEDOT:PSS		Direct laser writing and inkjet printing techniques	Biomedical applications	High power density	Massetti et al. (2020)
			Drop-casting		High deformability	Hasan et al. (2022)
	Porous PDMS		Combined with Bi_2_Te_3_-based thermoelectric legs		Enhanced power density	Toan et al. (2021)
	PEI doped carbon nanotube yarn		Sewed into a spacer fabric	Sensing of pressure and temperature	High temperature and pressure sensitivity	Zheng et al. (2022)
	CNTs/PEDOT:PSS and PEI-doped CNTs/PVDF		Simple laminated construction	Detection of light intensity	Excellent flexibility and outstanding durability	Zhang et al. (2022b)
	Bi_0.5_Sb_1.5_Te_3_ and Bi_2_Se_0.5_Te_2.5_		A sacrificial layer-assisted soldering fabrication method	Temperature detection	High output performance	Shi et al. (2022)
	CNT/PEDOT:PSS		Electrospinning and self-assembly strategy	Motion detection	High stretchability and seamability	He et al. (2022)

Self-supply power is a key element for achieving portability of wearable biosensors. The best solution is to abandon traditional external power sources and to allow the biosensors to be self-powered (Reid and Mahbub, 2020). In recent years, soft wearable electronic sensors based on nanomaterials have progressed significantly, benefited by hybrid nanomaterials such as carbon nanomaterials, metallic nanomaterials, metallic compound nanomaterials, and hybrid nanomaterials. As a result, the range of applications has considerably widened, for example, personal health monitoring, human-machine interfaces, smart tactile artificial skin, and personal prosthetics (Liu and Zhu, 2023). Due to the improvements in new nanomaterials, nanogenerators (NGs) can be fabricated with excellent mechanical flexibility and environmental adaptability. For instance, a triboelectric nanogenerator (TENG) can convert the mechanical energy obtained from the environment into effective electric power and signals (Rahman et al., 2022a). According to the capability of triboelectric nanogenerators, artificial intelligence technologies will greatly benefit, including fields such as machine learning, big data processing, and massive sensors with complicated network. In particular, combining the latest 5G technologies with artificial intelligence will allow the Internet of Things (IoT) to progress with great speed, emerging into a new era of human-machine interface (HMI) by applying self-powered operation based on TENGs (Yun et al., 2020). Advancing chemical sensor technologies will enable a generational update to integrated wearable system in a new era of IoT. IoT toward multifunctional, self-powered, and wearable chemical sensors with point-of-care testing (POCT) is an inevitable trend (Wen et al., 2020).

The published self-powered technologies mainly involve two typical types: self-powered sensors and self-powered integrated sensing systems (Ouyang et al., 2021; Tiwari et al., 2022). Self-powered sensors directly transform physical, chemical, or biological fluctuations from the surrounding environment/organisms into electrical information, while self-powered integrated sensing systems need to harvest energy and store it before utilizing it to power the integrated sensor. Both self-powered types of technologies have benefitted from the latest advances in materials science and device designs (Dai et al., 2021). For instance, Panda et al. (Panda et al., 2022) summarized numerous up-to-date piezoelectric biomaterials and device designs applied to Piezoelectric Energy Harvesters (PEHs) to harvest mechanical energy, e.g., motion from human movements and vibration from vital organs, and transform this harvested energy into electricity based on the piezoelectric effect, revealing a new possible way for health monitoring, especially organ health status.

Energy conversion is essential for self-powered technology, determining the power outputs, sensitivity, and durability of the sensor devices (Chadha et al., 2022). In this review, the latest progress on energy conversion strategies for biosensors and biomedical applications is discussed in detail, including sensors self-powered by piezoelectricity, triboelectricity, biofuel cells, photovoltaics, thermoelectricity, and others. Furthermore, this review highlights the state-of-the-art research in self-powered technology and discusses the potential applications and future trends of integrated self-powered biosensor systems.

## 2 Self-powered technology

Self-powered technologies allow sensing devices to extract and utilize energy from chemical/physical changes in the surrounding environment. Compared to a traditional power source such as a rigid primary or rechargeable battery, the characteristic strengths of self-powered technologies are their excellent deformability and wearability, high security, eco-friendly nature, and high economic efficiency (Prunet et al., 2021; Wu et al., 2021). This section introduces the latest research on self-powered technologies based on a variety of main strategies for energy conversion toward wearable biosensors and biomedical applications.

### 2.1 Self-powered by piezoelectricity

Composite-based piezoelectric nanogenerators (PENGs) can generate a potential difference when undergoing mechanical deformations, namely, the piezoelectric effect, and can then convert from mechanical energy to electrical energy. PENGs are widely adopted in wearable biosensors and biomedical applications as a prominent self-powered technology (Yu et al., 2022a). To cope with the intrinsic flexibility and various deformation of different human body parts, PENGs, or the sensing system integrated with PENGs, are required to be deformable and stretchable (Zhou et al., 2020). Previous research demonstrates that new polymer-based PENGs, advanced piezoelectric materials, and rational stretchable design can effectively contribute to wearable high-power-density PENGs and related integrated systems (Ng et al., 2022; Yue et al., 2022; Zhu et al., 2022).

**FIGURE 1:**
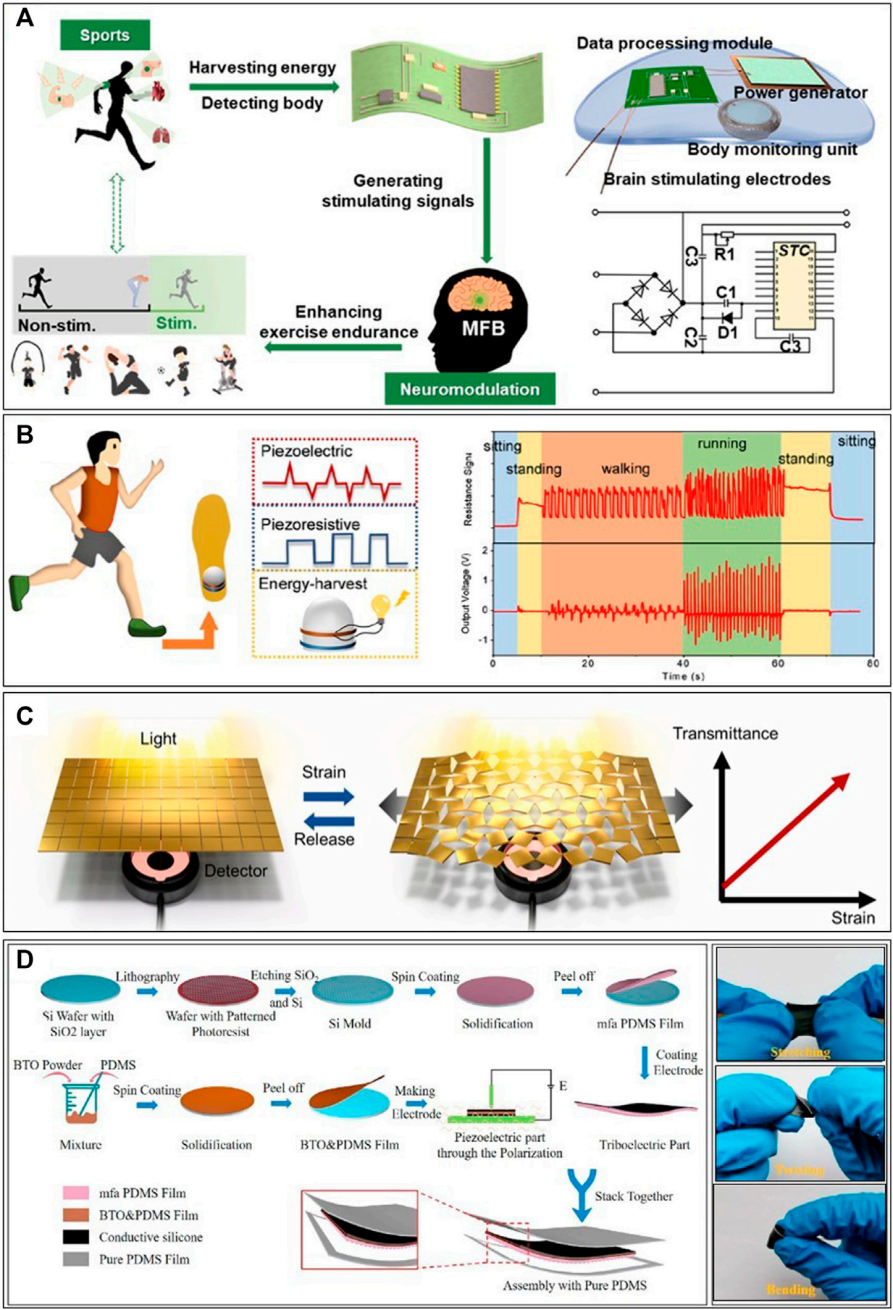
**(A)** Schematic illustration of the wearable and real-time brain-machine-interface system for enhancing exercise endurance showing the four parts of the system and the electronic circuit of the data processing module (Liang et al., 2022). **(B)** Schematic diagram of the dual-mode device incorporated into the insole to detect human motion *via* voltage/resistance variation of the dual-mode sensor during a series of body movements (Kong et al., 2022). **(C)** Schematic illustration of the GDE-R (the rotating square pattern) based piezo-transmittance strain sensor (Gu et al., 2021). **(D)** The fabrication process of the P-TPS and optical photos of the P-TPS under different deformations (Yu et al., 2022b).

PENGs have a wide range of biomedical applications, including personalized recognition and human-machine interfaces, owing to their high feasibility in terms of mechanical energy harvesting and flexible structure design (Askari et al., 2022; Zhou et al., 2022). Kang et al. (Kang et al., 2022) reported a ZnSnO_3_-surface-modified piezoelectric material (poly (vinylidene fluoride-co-trifluoroethylene)-based nanofiber) and judiciously designed a system-level piezoelectric device. This work demonstrates the device’s excellent piezoelectric properties for harvesting high-power energy (97.5 V and 1.16 μA) and even for sensing the imperceptible pulse in the arteries of the posterior tibial. Li et al. (Li et al., 2022) fabricated a highly flexible piezoelectric motion sensor, which is based on the promoting aid of polythiophene in the transformation process from α-phase polyvinylidene fluoride (PVDF) to β-phase PVDF. Furthermore, PENGs as a power supply for a brain-machine-interface platform have been developed recently. Liang et al. (Liang et al., 2022) proposed a novel wearable body-detecting/brain-simulating system self-powered by a flexible piezoelectric power generator. The whole system includes PENGs, body monitoring unit, data processing unit, and brain-stimulating electrodes, which are integrated into a flexible substrate (Figure 1A). This work reveals that the endurance performance of running mice is enhanced with the brain-stimulating electrodes of the system.

Most piezoelectric sensors suffer from a lack of robust and reliable sensitivity for various mechanical stimuli from rough or spiky surfaces of a subject, and experimental results indicate that improvement of the sensing configuration of pressure sensors could be an efficient coping strategy (Sharma et al., 2022). Kong et al. (Kong et al., 2022) prepared a self-protective pressure sensing device with a piezoelectric-piezoresistive dual mode (Figure 1B), based on a positive Poisson’s ratio elastic cylinder, which could significantly amplify the external pressure thereby enhancing the sensitivity and energy conversion efficiency of the fabricated piezoelectric device. Gu et al. (Gu et al., 2021) constructed a self-powered soft strain sensor based on the piezo-transmittance of an auxetic-pattern negative Poisson’s ratio elastomer. Specifically, the soft sensor uses a light-blocking Au-coated elastomer film, designed as a rotating square structure pattern. The measuring mechanism of the strain sensor is based on the changes in optical transmittance, correlating with changes in the gaps in the auxetic structure under externally applied strains (Figure 1C). The novel design endows the sensor with ultralow stiffness, high uniformity, fast response, and great robustness. Moreover, a self-powered wireless sensing system was successfully fabricated by integrating the developed strain sensor with a Bluetooth Low Energy 4.0 transceiver and a commercial solar cell, which effectively monitors device-structure health and human-body motion in real time.

Apart from this, PENGs incorporated with other types of energy conversion strategies, such as triboelectric nanogenerators, demonstrate excellent synergies with outstanding stretchability, high sensitivity, and excellent output performance (Zhang et al., 2021c). Kim et al. (Kim et al., 2021) developed a flexible piezoelectric-thermoelectric hybrid generator (f-PTEG) composed of a piezoelectric unit of poly (vinylidene fluoride-co-trifluoroethylene) film and a thermoelectric unit of bismuth telluride-based alloy blocks. Owing to the combined piezoelectric and thermoelectric effects, the hybrid f-PTEG could well integrate the energy converted from human body heat and movement. Pan et al. (Pan et al., 2022) constructed a transparent piezoelectric-photovoltaic energy harvesting device using the SnO-TiO_2_ quantum dots (QDs)/ZnO PN junction. Owing to the high quantum yield and appropriate potential of TiO_2_ QDs, the injection and separation of charge carrier are accelerated. The energy harvesting device exhibits high transmittance and obvious photovoltaic conversion enhancement, and can be an outstanding candidate in smart windows. Lee et al. (Lee et al., 2022) designed novel piezoelectric-triboelectric hybrid nanogenerators (PTHNG) based on MAPbBr_3_ (methyammonium lead tribromide) single crystals embedded into porous PVDF (MAPbBr_3_-PVDF), which simultaneously functions as a piezoelectric layer for a piezoelectric nanogenerator and as a dielectric layer for a triboelectric nanogenerator. The power density of the PTHNG is approximately 200 times larger than that of the pure PENG based on the same materials. Yu et al. (Yu et al., 2022b) proposed a stretchable self-powered piezo-triboelectric press sensor (P-PTS) fabricated by combining a piezo-film with a triboelectric nanogenerator (Figure 1D). Owing to the coupling of the piezoelectric effect with triboelectrification, the flexible-polymer-based P-PTS demonstrates satisfactory electrical sensitivity (voltage of 0.3 V kPa^−1^ and 4.3 nA kPa^−1^ under a pressure range of 0–200 kPa), excellent skin conformality, and considerable stretchability (∼187.32%). The output performances of the P-PTS are highly robust and almost unaffected after a series of various external forces. Moreover, the self-powered P-PTS is practical for monitoring a wide range of joint motion, such as different motion types of the knuckle, elbow, and wrist. The design concept of cooperation between different energy conversion strategies in this work is expected to promote advanced self-powered technologies in future follow-up research.

### 2.2 Self-powered by triboelectricity

Triboelectricity is delivered by highly efficient triboelectric nanogenerators (TENGs) *via* the process of friction between various materials with different triboelectric polarities. TENGs have gained enormous attention and numerous investigations on them have been performed in recent years, especially focusing on their applications in wearable biosensors and biomedical applications (Zhou et al., 2021). As an attractive self-powered technology, the merits of TENGs, and the related integrated systems, are outstanding, including low cost, a wide variety of available materials, the potential of harvesting wind energy, and tidal energy (Zhao et al., 2022a; Yuan et al., 2022). However, the practical applications of TENGs are still limited by insufficient stretchability, poor output performance, and a lack of reliable power management tactics.

**FIGURE 2:**
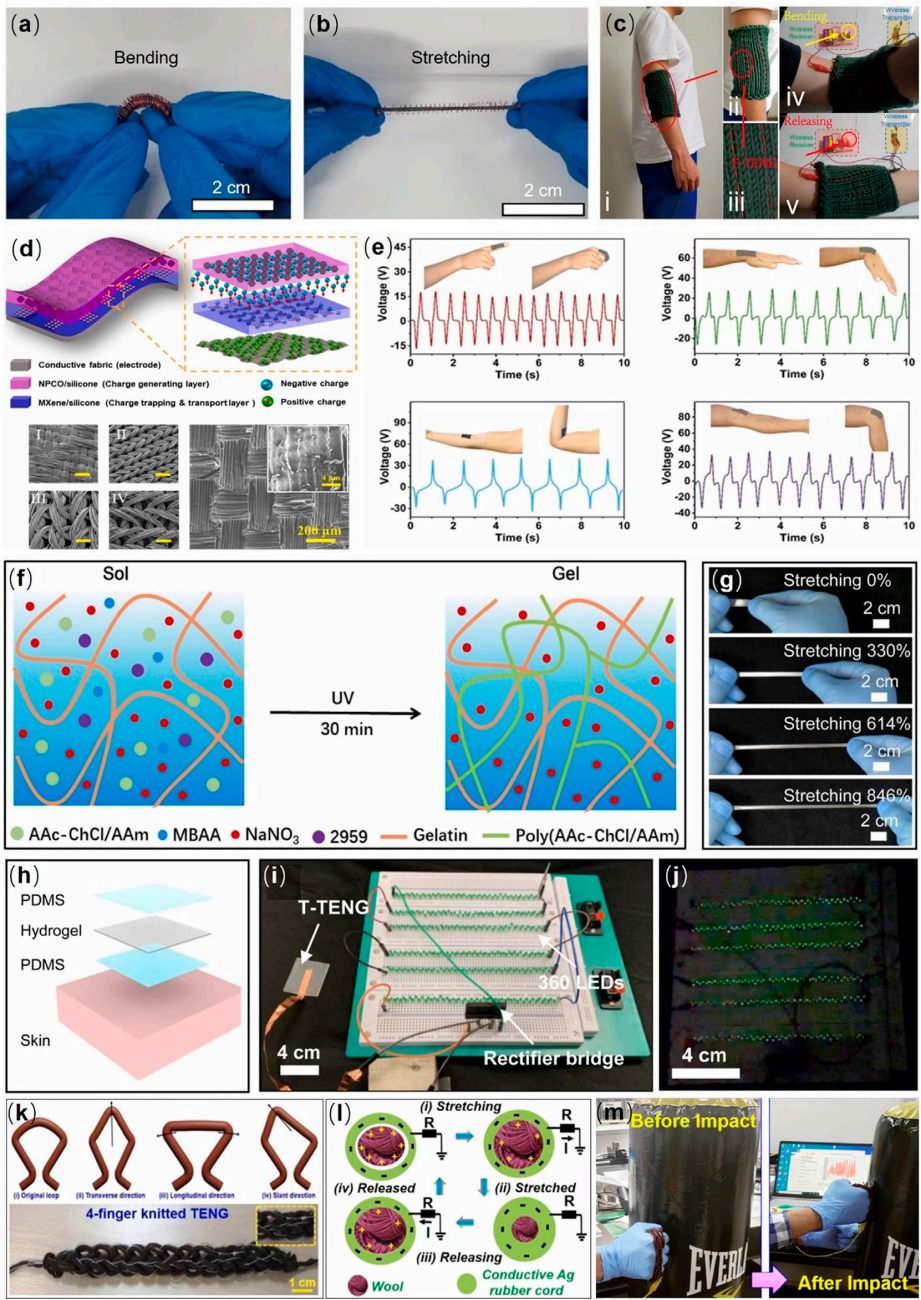
**(A)** F-TENG under bending. **(B)** F-TENG under stretching. **(C)** Smart clothing integrated with F-TENG (Zhao et al., 2022b). **(D)** Schematic description of the NDL-TENG. **(E)** Produced voltage signals of the self-powered biosensor integrated with NDL-TENG in response to the bending of different body joints (Rahman et al., 2022b). **(F)** Schematic illustration of the transparent, stretchable, and conductive hydrogel (TSCH). **(G)** Optical photos of the stretchability testing of TSCH. **(H)** Schematic illustration of the T-TENG based on hydrogels. **(I)** Optical photo of the setup for lighting LEDs using the T-TENG **(J)** Optical photos of the 360 LEDs lit by the T-TENG (Liu et al., 2022a) **(K)** Deformation of the knitted textile and optical photos of the 4-finger knitted TENG **(L)** Schematic illustration of the working mechanism of the developed F-TENG **(M)** Optical photos of an individual imparting a punch on a boxing kit before and after impact (Sahu et al., 2022).

In recent years, specialists mainly focused on chemical/physical modification and charge enhancement tactics to promote further improvement of self-powered wearable biosensors with TENGs (Wan et al., 2021; Peng et al., 2022). Qu et al. (Qu et al., 2022) designed a flexible multi-fingerprint-shaped wearable triboelectric tactile sensor (FTTS) with respectively independent fingerprint-shaped channels filled with eutectic gallium-indium liquid metal. Zhao et al. (Zhao et al., 2022b) constructed a self-powered stretchable biosensor with fiber-based triboelectric nanogenerators (F-TENG) for real-time body monitoring. The ultra-stretchable Ecoflex fiber and the design twining varnished wires endow F-TENG with excellent stretchability (∼600%) (Figures 2A, B). During practical application, F-TENG can be integrated into smart clothes and incorporated with wireless communication (Figure 2C), exhibiting a high potential to act as a promising alternative for next-generation biosensing systems.

Recently, a novel multifunctional double-layered triboelectric nanogenerator (NDL-TENG) was successfully developed by incorporating nanoporous cobalt oxide (NPCO) and Mxene into silicone (Figure 2D) (Rahman et al., 2022b). Combining the advantages of a high specific surface area and chemical oxygen functional groups, the NPCO effectively enhances the electronegativity and improves the triboelectric performance fourfold. At the same time, the Mxene in the silicone has the excellent ability to trap and transport charge. Compared to the pristine silicone tribo-layer, the Mxene/silicone efficiently increases the surface potential of NDL-TENG more than ninefold. Moreover, various commercial fabrics were constructed on a layer of NPCO/silicone to build up high stretchability and robustness. The optimized output performance of the designed NDL-TENG is excellent, including a high power density of 10.4 W m^−2^, an outstanding sensitivity of 5.82 V kPa^−1^, and a stretchability of ∼230%. Attached to various body joint parts, the NDL-TENG shows clear and stable physiological signals (Figure 2E), exhibiting its practical potential in medical rehabilitation applications.

For visual monitoring of the real-time condition of the detection subject, transparency is a crucial characteristic for TENGs toward functionalized wearable biosensors and biomedical applications (Wu et al., 2022a; Shahbaz et al., 2022). Yang et al. (Yang et al., 2022a) reported a stretchable polyvinyl alcohol/phytic hydrogel-based transparent triboelectric sensor for a self-powered medical human-machine interaction (HMI) sensing system. In the practical application of medical diagnosis, the HMI sensing system shows excellent mechanical durability (over 20,000 cycles) and considerable electrical performance (up to 1.33 W m^−2^), providing real-time assistance for both clinicians and patients. Liu et al. (Liu et al., 2022a) fabricated a flexible and transparent triboelectric nanogenerator (T-TENG) based on a self-developed conductive hydrogel layer and adopted polydimethylsiloxane (PDMS) layers as packaging (Figures 2F, H). The T-TENG demonstrates a highly flexible nature (∼850%) (Figure 2G), high transparency (∼90%), and outstanding electrical performance (open-circuit voltage of ∼684.4 V and short-circuit current of ∼116.5 μA per 16 cm^2^). The output performance of the T-TENG is quite stable under various conditions, which include being folded, twisted, smashed, and stretched over 200 cycles, and it supplies sufficient power for 360 LEDs (Figures 2G, I, J). Furthermore, the T-TENG mounted on the fingers outputs clear electrical information along with information on the body’s mechanical movement and deformation, demonstrating the high sensitivity of the self-developed T-TENG and its potential for effective energy harvesting.

Owing to the intercoupling of triboelectrification with electrostatic induction, TENGs show considerable potential for converting irregular and randomly distributed energies into consistently usable electricity. Zeng et al. (Zeng et al., 2022b) introduced a self-powered rain droplet sensor using a stimulating liquid-solid triboelectric nanogenerator (LS-TENG) to harvest water/raindrop energy. The LS-TENG demonstrates high sensitivity to contact with surrounding water motion, generates an electrical signal in response to the droplet impact force on the LS-TENG and the electrostatic induction of water flowing down the fluorinated ethylene propylene film, and harvests irregular energies from the ambient environment to sustainably supply power for portable electronics. Additionally, the LS-TENG can also sense the falling speed and velocity of water droplets. This work sheds valuable light on the improvement of the conversion effectiveness of irregular energies based on TENGs toward wearable biosensing systems.

Moreover, TENGs also demonstrated their potential in the reuse of a large number of waste textiles generated due to the lack of public recycling awareness. Sahu et al. (Sahu et al., 2022) proposed recycling abandoned worn-out textiles into single-electro-mode triboelectric nanogenerators for self-powered devices (Figure 2L). The positive and negative triboelectric layers adopted various waste textiles. A triboelectric nanogenerator (TENG) was fabricated from the fibers of a cigarette butt by the 4 fingers knitted technique (Figure 2K) and could deliver a peak-to-peak voltage of 4.2 V and a current of 2.7 nA under mechanical stretching and releasing. The newly designed triboelectric nanogenerator can harvest biomechanical energy from various sports by attaching it to sports accessories such as socks and exercise bands (Figure 2M). Moreover, combining the self-powered sensor with digital signal processing (e.g., wavelet transformation and wavelet packet transformation) can effectively promote intelligent sports devices.

### 2.3 Self-powered by biofuel cells

Biofuel cells (BFCs), as a green self-powered strategy, can convert the biochemical energy of the components contained in various body fluids (biofuel, e.g., lactate and glucose) into available electrical energy through biocatalytic reactions. BFCs have many prominent advantages, such as environmental friendliness, easy integration, and good biological compatibility, which contribute to their broad use in self-powered on-body biosensors and biomedical applications (Dutta et al., 2022). However, BFCs still suffer from a low power output and non-ideal operational stability in the long term. An effective approach to solve these obstacles is the exploration of different nanocatalytic systems based on various advanced enzymes (Gu et al., 2022), nanomaterials (Haque et al., 2023), and energy management (Assad et al., 2022). A selective self-powered dibutyl phthalate aptasensor was prepared based on a flexible enzymatic biofuel cell (EBFC) using a glucose oxidase/Bi_3_Ti_2_O_8_F/carbon nanotube-reduced graphene oxide (CNTs-rGO) layer as the anode and a layer of aptamers fixed with AuNPs/CNTs-rGO as the corresponding cathode (Qin et al., 2022). Owing to the modification of Bi_3_Ti_2_O_8_F with catalase mimicking properties on the anode, the output performance of the EBFC effectively improves. Yang et al. (Yang et al., 2022b) prepared an advanced lactate oxidase bio-anode modified with multi-walled carbon nanotube (MWCNT)/naphthoquinone, which enhances the electron transfer efficiency of BFCs. Compared to previously published flexible BFCs, the fabricated bio-anode-based biofuel cell is competitive in terms of power-density output. Among the advanced nanomaterials for enzyme immobilization, Xu et al. (Xu et al., 2022a) proposed a coral-like hierarchical meso-macroporous carbon (CHMC) prepared from cucumber, which facilitates the high-quality immobilization of enzymes (LOx) and accelerates the transfer of electrons in the bioelectrodes. Furthermore, a CHMC-based lactate/air BFC was constructed to harvest energy from practical samples. This work indicates a novel avenue for the synthesis of advanced bioelectrode materials for BFC-based healthcare applications.

**FIGURE 3:**
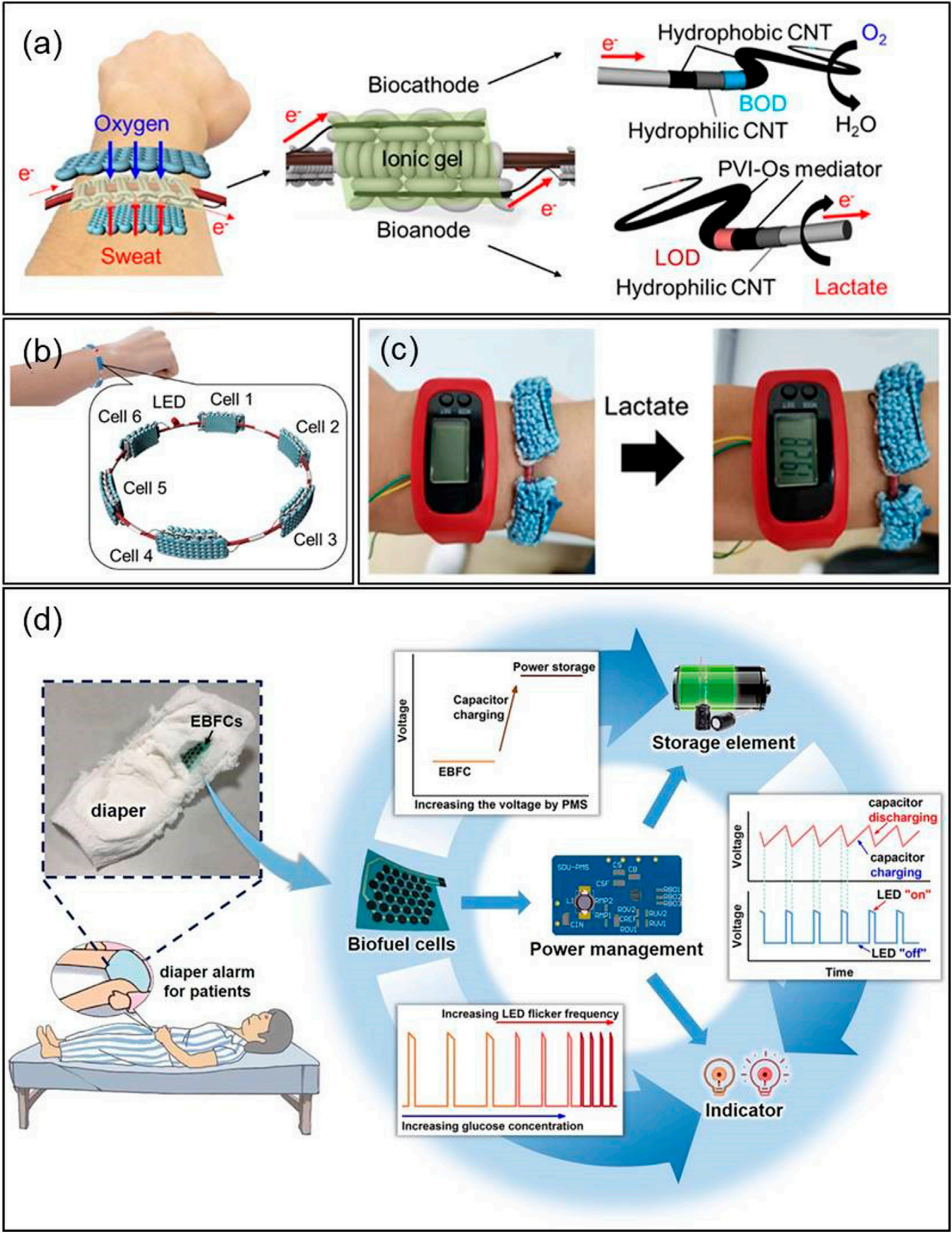
**(A)** Schematic diagram of the fiber-crafted biofuel cell based on lactate biofuel from human sweat. **(B)** The configuration of the six-cell bracelet. **(C)** Optical photos of powering an electronic watch with the bracelet-type biofuel cells (Yin et al., 2021). **(D)** Schematic diagram showing the components of the diaper alarm integrated with the biofuel cell-type biosensor and the circuit diagram of the alarm device (Zhang et al., 2021d).

The rational design of biosensing systems integrated with BFCs would provide not only high efficiency but also considerable assistance for medical staff and disabled patients (Wang et al., 2022a). Yin et al. (Yin et al., 2021) reported a high-power lactate-based biofuel cell using a hydrophilic supportive textile woven from fibers to store sweat (Figure 3A). To boost the output performance, series-connection anode/cathode fibers are bundled and wrapped around the wrist in a bracelet-type biofuel cell (Figure 3B). The six-cell bracelet produces power at 2.0 V to sufficiently drive digital wrist watches (Figure 3C). Su et al. (Su et al., 2022) developed a low-cost wearable sensing system composed of an electrochemical biosensor integrated with a diaper and a smartphone with a data processing application (App). The multifunctional sensing system not only reminds the nurse to change the diaper but also monitors the real-time health status of patients. Apart from the App alarm, Zhang et al. (Zhang et al., 2021d) fabricated an innovative biofuel cell-type biosensor system incorporated into the diaper to detect information about urine glucose levels for diabetic patients, using an LED flashing alarm (Figure 3D). The system was self-powered by its biofuel cell module, with glucose oxidase as the bioanode and MnO_2_ as the biocathode, and, at the same time, the power density (up to 220 μW cm^−2^) generated by the biofuel cell was stored in a capacitor to power the LED flash. The glucose concentration was proportional to the LED’s flashing frequency, associated with the charging rate of the capacitor, and the corresponding linear correlation is excellent in the range of 1–5 mM glucose concentration. During the anti-interference experiment using uric acid and carbamide, the device was almost unaffected by the interferences.

**FIGURE 4:**
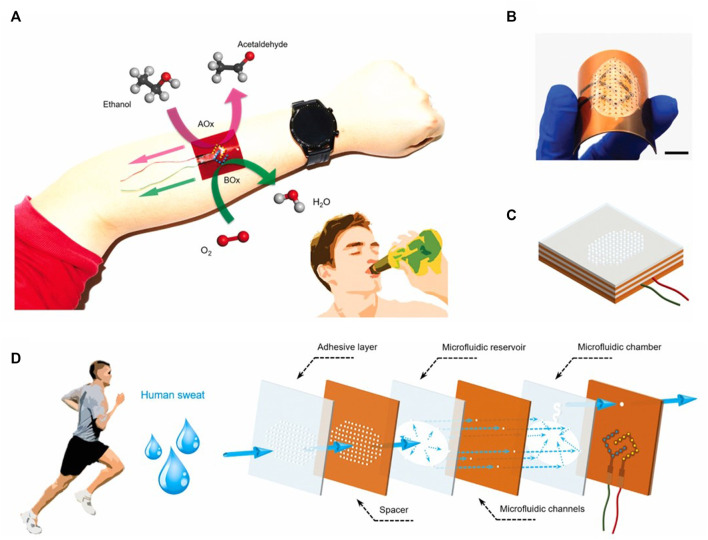
**(A)** Schematic illustration of a flexible and wearable epidermal ethanol BFC that harvests bioenergy in real-time from the perspiration of individuals after drinking alcohol. **(B)** Optical photo of a flexible epidermal ethanol BFC (scale bar, 1 cm). **(C)** Schematic illustration of an epidermal ethanol BFC. **(D)** Schematic illustration of the microfluidic module for continuous real-time *in situ* fresh sweat sampling, transfer, storage, and excretion on human skin (Sun et al., 2021).

Different from utilizing human endogenous substances to generate energy in previously reported research, Sun et al. (Sun et al., 2021) proposed a noteworthy self-powered epidermal ethanol-oxygen biofuel cell based on exogenous substances in the perspiration of individuals after drinking alcohol (Figure 4A). The epidermal BFC includes two functional modules, namely, a specially designed microfluidic module for the collection and transport of the sweat, and a BFC module for harvesting bioelectricity in real-time (Figures 4B, C). Additionally, the two modules use highly flexible PI and PET as substrates to ensure skin-conformal adhesion of the wearable epidermal BFC in practical applications (Figure 4D). This work enlarges the potential circumstance of the BFC for on-body bioelectricity harvesting and conversion.

### 2.4 Self-powered by photovoltaics

**FIGURE 5:**
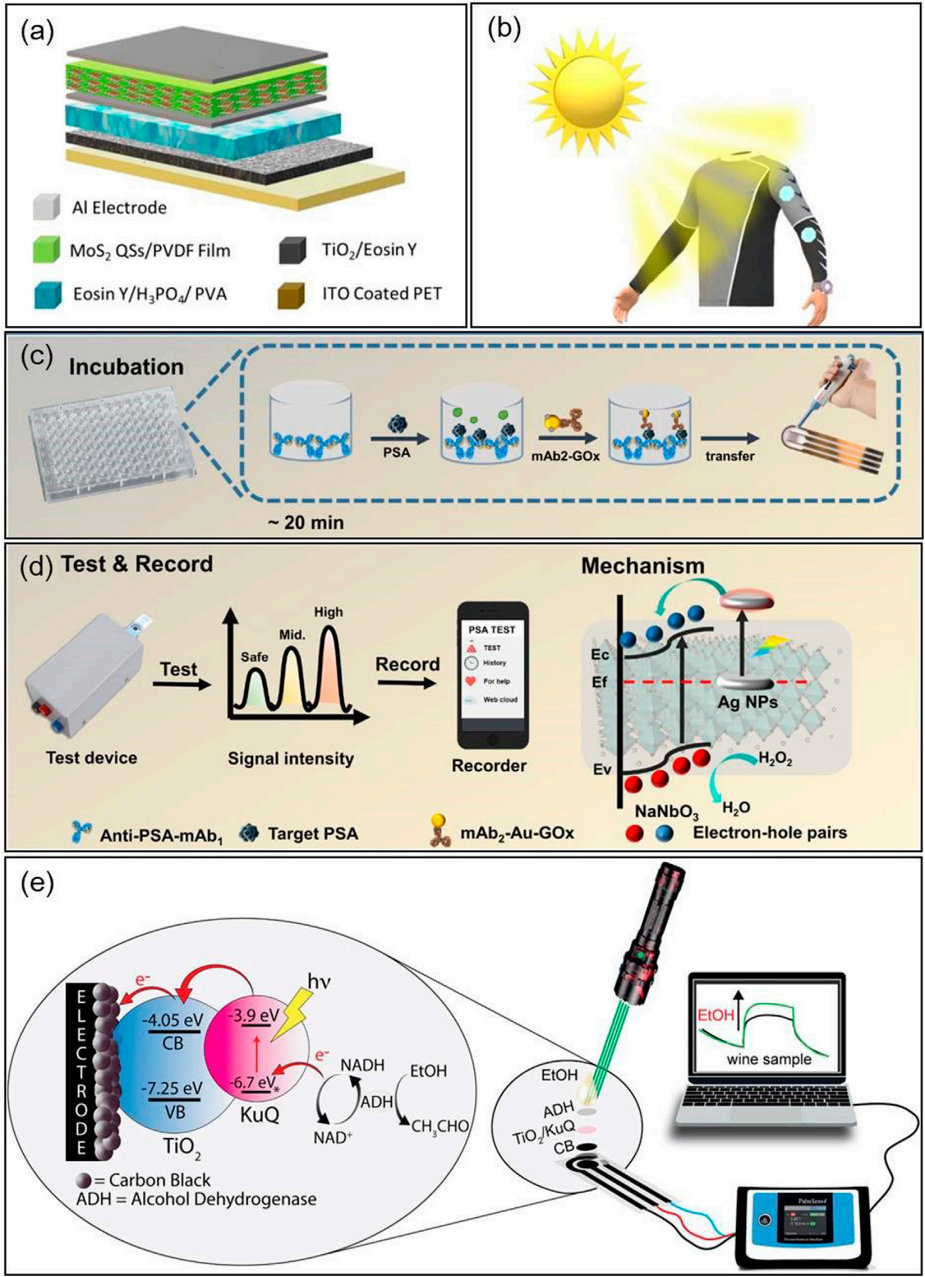
**(A)** Schematic diagram of the PSCPC device. **(B)** Conceptional representation of the PSCPC absorbing natural sunlight for powering smart gadgets (Nardekar et al., 2022). **(C)** Schematic illustration of the incubation process of target PSA in a microtiter plate. **(D)** Schematic illustration of the PEC recording platform for 3D printed microdevices and the photoelectron transfer of the Ag/NaNbO_3_ composites. (Xu et al., 2022b). **(E)** Schematic illustration of the photoelectrochemical sensing system and the experimental set-up for detection of ethanol (Mazzaracchio et al., 2022).

Owing to years of market inspection and technical improvement, solar photovoltaics is the most mature and viable self-powered technology among the up-to-date emerging self-powered strategies in the field of wearable biosensors and biomedical applications (Kumari et al., 2022). To cope with the requirement of miniaturization and high-power density for a wearable biosensor, researchers have devoted enormous efforts to developing advanced multifunctional materials and rational device configurations for photoelectrochemical biosensors (Liu et al., 2022b; Wu et al., 2022b; Jahromi et al., 2022). Nardekar et al. (Nardekar et al., 2022) reported a self-developed photovoltaically self-powered cell (PSCPC) based on a dual-functional nanocomposite film composed of a two-dimensional quantum sheet of molybdenum disulfide, which is incorporated into a polyvinylidene fluoride (PVDF) layer to improve the dielectric properties of pure PVDF (Figure 5A). The output performance of the novel piezoelectric nanogenerator fabricated using the MoS_2_ QSs-PVDF film is remarkable, with a high output voltage (47 V_pp_) and power density (3.2 mW m^−2^). Due to the significant role of the TiO_2_ intermediator, the PSCPC delivers an excellent charging voltage (900 mV) and a remarkable photocurrent (25 μA). Furthermore, when practically integrated with clothing (Figure 5B), the unique PSCPC can sufficiently supply power for flexible wearable electronics, with energy scavenging from natural sunlight and ambient indoor light. This work demonstrates an effective design strategy for combining smart biosensors with photovoltaic materials in the field of converting and storing energy.

Researchers have also put great efforts into the elemental doping/co-doping strategy to reasonably tune bandgaps in order to effectively facilitate the photovoltaic effect and to improve the photoelectrochemical response in self-powered biosensing systems (Ai et al., 2022; Ma et al., 2022). Cheng et al. (Cheng et al., 2019) constructed a novel nitrogen-doped carbon quantum dot (NCQD)-based photoelectrochemical (PEC) biosensor driven by visible light for the detection of organophosphate pesticides. The excellent optical performance of the NCQDs, including a narrow band gap, fast electron transportation, and upconversion photoluminescence, endow the NCQD/TiO_2_/ITO electrode with a high photocurrent response, which is approximately 42 times that of the TiO_2_/ITO electrode under visible light. Gao et al. (Gao et al., 2022) designed Fe^3+^-Li^+^ co-doped Zn_1-2x_ (Fe_x_Li_x_)O films using an economical sol-gel method. The Zn_1-2x_ (Fe_x_Li_x_)O films demonstrate an anomalous photovoltaic effect with high performance (a switchable photoresponse of 34.1 mA W^−1^ and a power conversion of 10.3%), which is attributed to the enhancement of current density and fill factor in the fabricated films. To explore novel ferroelectric photovoltaic materials with a narrow band gap, Wang et al. (Wang et al., 2022b) proposed an advanced 0.8NaNbO_3_-0.2La(Mn_0.5_Ni_0.5_)O_3_ (0.2NLMNO) ferroelectric ceramic fabricated by a solid-state process. The doped La(Mn_0.5_Ni_0.5_)O_3_ can induce a crystal structure with higher symmetry and can significantly decrease the resistance between grains and grain boundaries. The photocurrent response of 0.2NLMNO is approximately 10 times higher than that of pure NaNbO_3._

Furthermore, the light source and photoelectrochemical active species are both crucial factors for photoelectrochemical biosensors that are based on a measurable electrical signal enhanced by photoelectrochemical active species under illumination (Yang et al., 2020; Tang et al., 2022). Xu et al. (Xu et al., 2022b) presented a UV light-driven photoelectrochemical (PEC) biosensor based on the co-enhanced effects of localized surface plasmon resonance and portable piezoelectric effect between Ag nanoparticles and NaNbO_3_ nanomaterials (Figure 5C). The biosensing platform integrated with the fabricated PEC biosensor exhibits a wide linear range and low detection limits for the cancer marker PSA in point-of-care testing (Figure 5D). Mazzaracchio et al. (Mazzaracchio et al., 2022) developed an economical self-powered photoelectrochemical biosensor based on a unique screen-printed electrode with carbon black (CB) as a conductor. The TiO_2_/KuQ is homogeneously dispersed on the surface of the CB layer and expands the range of absorbable light from the UV to the visible light region (Figure 5E). The photoelectrochemical biosensor exhibits high analytical performance, with an excellent linear range (between 50 μM and 8 mM) and an outstanding detection limit (∼20 μM) for NADM measurement. The sensor incorporating alcohol dehydrogenase can detect ethanol in white wine samples at a competitive detection limit (∼0.062 mM), which sheds valuable light on the practical application of other dehydrogenase enzymes in the field of biosensors based on screen-printed-electrodes. To avoid the possible damage of biological subjects caused by UV light, Zhai et al. (Zhai et al., 2019) constructed a near-infrared light-triggered photoelectrochemical biosensor for the ultrasensitive detection of alfa-fetoprotein (AFP), which is based on the synergistic effects of ZnO, CdS, and upconversion luminescence of NaYF_4_:Yb and Tm nanophosphors. The fabricated biosensor presents an ultra-wide linear range (0.01 ng mL^−1^) and an ultra-low detection limit (5 pg mL^−1^) for the target AFP. The upconversion-nanophosphor-based PEC biosensor provides an attractive alternative for testing other cancers in practical clinical analysis.

### 2.5 Self-powered by thermoelectricity

Thermal gradient-based thermoelectricity is a promising source of energy for powering wearable biosensors and epidermal healthcare applications (Nozariasbmaez et al., 2020; Sun et al., 2022). When there is an uneven distribution of temperature between n-type and p-type thermoelectric materials, the charge carriers (electrons and holes) will migrate from the relatively high temperature region to the low temperature region. The efficiency of converting heat into electricity is correlated with temperature difference (ΔT) and the ZT value (equals to S^2^Tσ/к) of thermoelectric (TE) materials (Jaafreh et al., 2022; Zhi et al., 2022), which suggests explicitly viable approaches for designing rational thermoelectric generators (TEGs) with high energy conversion efficiencies.

**FIGURE 6:**
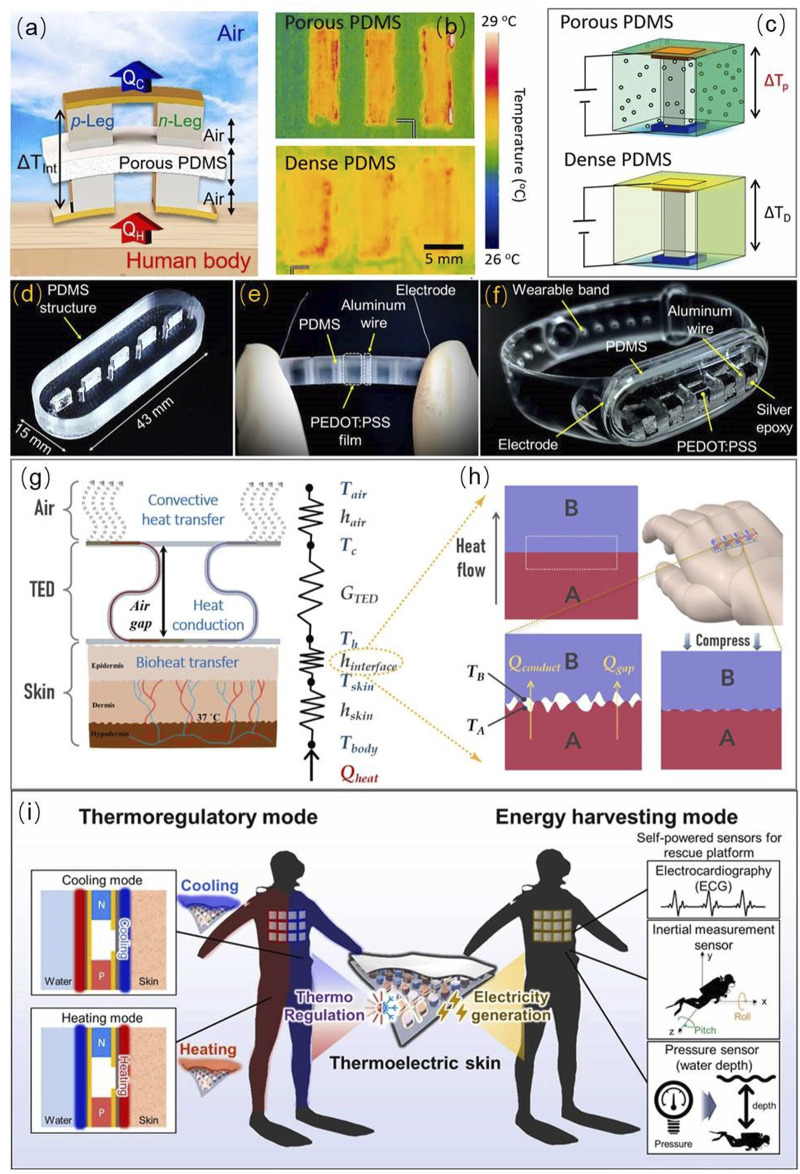
**(A)** Schematic diagram of a partially filled TEM. **(B)** Temperature response of the hot-side surfaces of pP TEM and dP TEM when direct current was used. **(C)** Schematics of the TEM configurations during the direct current experiments (Jung et al., 2021). **(D)** Optical photo of the fabricated PDMS structure, **(E)** side view of the wearable thermoelectric generator, and **(F)** a wearable band integrating the fabricated thermoelectric generator (Hasan et al., 2022). **(G)** Schematic diagram of the complete thermal process for a wearable S-TED. **(H)** Interface thermal resistance analysis (Lv et al., 2021). **(I)** Schematic illustration of dual modes (energy harvesting mode/thermoregulatory mode) that the TES switch between (Jung et al., 2022).

In terms of exploring excellent thermoelectric materials, significant effort has been made regarding organic/inorganic TE materials and optimization strategies for TE materials (Chen et al., 2021; Soleimani et al., 2021; Yang et al., 2022c). Recently, Toan et al. (Toan et al., 2022) developed an ultra-flexible considerable-performance thermoelectric generator (TEG) using a silicon rubber sheet and thermoelectric material fabricated by electrodeposition. The ultra-flexible TEG displays a high output performance, with an output voltage of 1 V and power density of 552.9 μW cm^−2^ under a ΔT of 60 K. Moreover, based on the ultra-flexible TEG, a self-powered temperature/humidity monitoring electronic device was successfully constructed. Massetti et al. (Massetti et al., 2020) prepared a novel micro-sized integral organic thermoelectric generator (µ-OTEG) using PEDOT: PSS and a doped fullerene derivative as the p-type and n-type legs, respectively, which are embedded into a plastic substrate using a fully direct writing technique. The reported flexible µ-OTEG exhibits an excellent power density (30.5 nW cm^−2^) and small thermal gradients, demonstrating the potential of scalable organic-based TEGs for low-power real life biosensing applications. Jung et al. (Jung et al., 2021) fabricated a new soft thermoelectric module (TEM) using a porous polydimethylsiloxane (PDMS) filler (pP filler) and Bi_2_Te_3_-based thermoelectric legs (Figure 6A). Due to the low thermal conductivity (0.8 W m^−1^ K^−1^) of the pP filler, the internal ΔT was increased by successfully suppressing thermal bypass. Compared to the reference TEM samples, containing dense PDMS filler and the pP filler, the partially filled TEMs presented a higher power density (123% that of the pP filler sample) and an excellent ZT value (0.75) (Figures 6B, C). Utilizing body heat, eight in-series partially filled TEMs can power 32 LEDs over 30 min. The design approach used in this work shows attractive potential in the field of stretchable self-powered biosensors.

The efficient structural configuration of flexible/stretchable thermoelectric devices has also garnered the significant attention of increasingly more researchers (Fan et al., 2021; Toan et al., 2021). Hasan et al. (Hasan et al., 2022) developed an efficient and wearable thermoelectric generator based on vertically aligned thermoelements comprised of a p-type PEDOT:PSS film and an n-type aluminum wire-based thermoelement. A wearable wrist-based thermoelectric generator, with five pairs of thermoelements, exhibited an excellent output voltage of 5.15 mV at a ΔT of 80 K (Figures 6D–F). Zheng et al. (Zheng et al., 2022) prepared an ingenious self-powered two-mode temperature and pressure sensor with tridimensional arrays achieved by sewing CNT yarn segmentally modified with polyethyleneimine into a high-arrangement-density spacer fabric. Zhang et al. (Zhang et al., 2022b) designed a novel flexible self-supported laminated thermoelectric device based on a rational array of thermal parallel and series electrical connections, using CNTs/PEDOT:PSS as p-type materials and CNTs/PVDF self-supported films as n-type materials. The obtained thermoelectric device generates an output voltage of 3.31 mV under a ΔT of 35 K and demonstrates promising potential in the detection of light intensity due to the prominent photothermal characteristics of CNT-based materials. To make better use of the vertical temperature gradient in the plane of the device, Lv et al. (Lv et al., 2021) proposed a flexible three-dimensional spring-shaped thermoelectric device (S-TED) based on dual elastomer layers with air gaps, showing excellent compressibility and effectively harvesting vertical waste heat (Figures 6G, H). The as-fabricated S-TED, using only three pairs of p-n couples, can deliver an excellent output power (749.19 nW) under a vertical ΔT of 30 K, which outperforms other reported flexible thermoelectric devices (416.22 nW cm^−2^). The design strategy of the S-TED opens up new possibilities for the utilization of human body heat in wearable biosensors and biomedical applications.

Innovations in fabrication techniques can also contribute to the enhancement of the wearable suitability of self-powered biosensing devices integrated with thermoelectric generators. Shi et al. (Shi et al., 2022) adopted a soldering fabrication process with the assistance of a sacrificial layer to fabricate a novel stretchable thermoelectric generator (STEG), comprised of kirigami-inspired flexible electrodes connecting 50 couples of cubic-shaped thermoelectric legs. In the work, various biomedical applications of the STEG were investigated and these show the potential of the as-fabricated STEG as wearable biosensing devices. He et al. (He et al., 2022) developed a CNT/PEDOT:PSS-based thermoelectric nanofiber yarn by integrated a coagulation-bath electrospinning technique with a self-assembly strategy, which can continuously produce high-stretchability (∼350%) and high-seamability nanofiber yarns. CNT/PEDOT:PSS is effectively loaded onto every fabricated nanofiber during the spinning process due to the phase separation and self-assembly effect. Biosensing platforms integrating the nanofiber yarns with gloves and masks can accurately identify cold/heat sources and sufficiently supply power for human respiration monitoring.

Thermoelectricity is also an excellent solution to address the practical conundrum of supplying power to wearable electronics in extreme environmental conditions for a long-term period, where bulky traditional batteries are not viable. Jung et al. (Jung et al., 2022) proposed a stretchable dual-function thermoelectric skin (TES) to harvest thermoelectric power and regulate the temperature of the subject wearing the TES underwater (Figure 6I). Due to the structure of the serpentine array and the reasonably designed backbone, the conductive elastomer-based TES demonstrated a high stretchability (up to 230%) and excellent mechanical durability. The TES generated a competitive power density (3.42 mV cm^−2^), the highest to date according to a comparison with published research on stretchable wearable thermoelectric devices. Furthermore, the dual-function TES units were integrated with the deformable surfaces of a neoprene dry-suit to validate their strength in practical applications. The sensors of the integrated suit could be sufficiently self-powered by the TES units to wirelessly monitor the real-time human body temperature, which was simultaneously regulated by the multiple TES units, based on a temperature feedback loop algorithm. The design concept and engineering principles of this work shed significant light on the future of underwater electronics.

## 3 Conclusion

Owing to their attractive potential in continuously non-invasive/real-time medical diagnosis, self-powered wearable biosensors have attracted enormous attention and efforts from researchers in various fields. In this review, the recent research advances in self-powered technologies toward wearable biosensors are discussed, mainly focusing on several typical self-powered strategies such as piezoelectric nanogenerators, triboelectric nanogenerators, biofuel cells, photovoltaically self-powered cells, and thermoelectric generators. The novel functional materials and stretchable structure designs in the above self-powered strategies are highlighted, which promote the advancement of self-powered wearable biosensors and the related integrated medical applications.

Over the past few decades, the number of investigations on wearable electrochemical sensors toward biomedical applications has increased. Nevertheless, some major obstacles need to be cleared to achieve the requirements for continuous health monitoring of body biomarkers over wide ranges in a reliable manner. Among these obstacles, the power supply units for most biosensors are not stable due to physical motions such as bending, twisting, and stretching. Therefore, a flexible and stretchable self-power unit is expected to provide a correct, reliable, and high-quality energy supply to ensure the continuous monitoring functionality of wearable biosensors. Moreover, insufficient output energy density remains a pivotal obstacle to be resolved for the broad practical biomedical application of self-powered biosensing systems. Further innovations in functional materials, stretchable device integration, and optimized energy management strategies are expected to lead to the fabrication of next-generation advanced biosensors with excellent self-powered ability, miniaturization, and intellectualization.

Overall, this review provides insights into and future trends of self-powered technologies based on nanomaterials toward wearable biosensors in biomedical applications, particularly for flexible, stretchable, and continuous health monitoring systems.
